# Biochemical Responses of Atacama and Blesbok Sweet Potato (*Ipomoea batatas* L.) Cultivars to Early Drought Stress

**DOI:** 10.3390/plants14223532

**Published:** 2025-11-19

**Authors:** Fikile N. Makhubu, Lebogang E. Siviya, Molemi E. Rauwane, Sunette M. Laurie, Ntakadzeni E. Madala, Sandiswa Figlan

**Affiliations:** 1 Department of Agriculture and Animal Health, University of South Africa, Florida Park, Roodepoort 1710, South Africa; 2 Department of Botany, Nelson Mandela University, Gqeberha 6031, South Africa; 3 Agricultural Research Council-Vegetable, Industrial and Medicinal Plants (ARC-VIMP), Pretoria 0001, South Africa; 4 Department of Biochemistry, University of Venda, Thohoyandou 0950, South Africa

**Keywords:** climate change, drought tolerance biomarkers, LC-MS, primary and secondary metabolism, sweet potato metabolomics

## Abstract

Sweet potato is a nutrient-dense crop with the potential to improve food security, yet its productivity is constrained by drought stress. Metabolic profiling in sweet potato, particularly in response to abiotic stress, remains poorly understood, with limited knowledge on the metabolites contributing to drought response. The study aimed to profile and compare metabolites in drought-tolerant (cv Atacama) and drought-susceptible (cv Blesbok) sweet potato cultivars under water-deficient conditions. The cultivars were grown in a rainout shelter during the 2024 growing season at the Agricultural Research Council-Vegetable and Industrial Medicinal Plant (ARC-VIMP). The trial was laid out in a randomized block design with a plot size of 242 m squared with three drought treatment conditions, i.e., 30%, 50%, and 70% field capacity (FC). After two weeks of drought stress imposition, leaf samples were collected and analyzed for metabolite changes using untargeted ultra-performance liquid chromatography-mass spectrometry (UPLC-MS). Using chemometrics analysis, mainly using principal component analysis (PCA) and orthogonal partial least squares discriminant analysis (OPLS-DA), significant separation was shown between the three drought stress conditions and the two cultivars, highlighting variable metabolic accumulation. Ten significantly regulated metabolites were identified (VIP > 1, *p* < 0.05), with the most pronounced log_2_ fold changes observed for kaempferol-3-O-galactoside (3.48), chlorogenic acid (3.34), glc-glc-octadecatrienoyl-sn-glycerol (3.14), and apigenin-7-O-β-D-neohesperidoside (2.71). Metabolite concentration varied in the two cultivars, although most were positively correlated with Atacama. Enriched pathways included flavonoid biosynthesis, zeatin biosynthesis, and starch and sucrose metabolism. These findings highlight cultivar-specific metabolic responses and propose candidate biomarkers for breeding drought-tolerant sweet potato.

## 1. Introduction

Drought stress remains a major limiting factor to crop production, negatively affecting plant growth and development at various stages [1,2]. Plants respond to drought stress through several complex biological mechanisms, including changes in metabolism, which are critical for maintaining homeostasis and ensuring survival under adverse conditions [3]. Metabolic adjustment involves both primary metabolites, which are essential for growth and development, and secondary metabolites, which play specialized roles in stress tolerance [4,5]. Under drought, plants often accumulate osmolytes and osmoprotectants as primary metabolites, alongside defense-related secondary metabolites, which together enable the plants to withstand the harsh conditions. Primary metabolites are particularly affected due to reduced CO_2_ assimilation, which alters absorption [6].

Sweet potato (*Ipomoea batatas* [L.] Lam, Convolvulaceae) is a widely cultivated staple crop with global production reaching 88.87 million tons in 2021, with China producing 53.6% of the total [7]. The crop is valued for its rich nutritional value, providing starch, dietary fiber, protein, and essential minerals such as manganese, copper, potassium, and iron [8]. It is also an important source of vitamins like B-complex, vitamin C, and E, along with provitamin A (carotenoids), anthocyanins, flavonoids, and coumarin [8,9]. Various sweet potato cultivars are cultivated all over the world, and they are distinguished by the different colors of their flesh and their unique phytochemical composition. The nutritional values and bioactivities of phytochemicals found in different plant species may vary intrinsically [10,11]. Also, consumer preference for traits is based mainly on appearance and taste [12]. The primary bioactive substances often found in sweet potatoes are glycosides, terpenoids, and polyphenols (mostly flavonoids and phenolic acids) [13].

Metabolomics is an important approach for identifying and analyzing metabolic phenotypes within intricate cellular processes [14]. While several sweet potato studies have focused on metabolite profiling [12,13,14,15,16,17,18,19], few have addressed drought stress. Yin et al. [3] reported significant molecular differences between the drought-sensitive (Jinong432) and the drought-tolerant (Zhenghong23) cultivars, highlighting the role of amino acid metabolism, respiratory pathways, and antioxidant systems in drought tolerance. Similarly, Zhou et al. [20] found 75 metabolites, including carbohydrates, amino acids, flavonoids, and organic acids, which were more abundant in the drought-resistant cultivar WZ56, as compared to the sensitive cultivar NZ2. Despite such advances, most metabolomic studies in sweet potato have focused on nutritional traits, with limited attention to drought stress responses. This gap is even more apparent given that metabolomic research on sweet potato is relatively new, having developed mainly in the last decade [21]. Notably, the few existing studies on drought-related metabolomics have largely focused on Asian cultivars, with minimal attention to Southern African genotypes. Consequently, there is a lack of understanding of how locally adapted sweet potato cultivars modulate their metabolic pathways under drought conditions, particularly during early stress. This gap limits efforts to identify drought-responsive biomarkers that can support breeding for climate-resilient sweet potato varieties. Therefore, the current study aimed to determine the biochemical responses of two sweet potato cultivars (drought susceptible and tolerant) in response to early drought stress. These cultivars were chosen based on their contrasting physiological responses to water deficit, as documented in previous field evaluations conducted in South Africa.

Atacama (CIP, Peru origin) is known for its drought tolerance and has consistently demonstrated stable yield under limited water availability, making it a suitable model for identifying stress-adaptive metabolic traits. In contrast, Blesbok (South African cultivar) is intermediately susceptible to drought and remains one of the most widely cultivated sweet potato varieties in South Africa due to its agronomic familiarity and local availability [22,23]. The selection of these two cultivars is also supported by their relevance to current climate conditions in Southern Africa, where prolonged dry periods increasingly threaten food production. Studying their metabolic profiles under controlled drought conditions thus offers valuable insight into cultivar-specific biochemical responses and provides a foundation for future breeding efforts targeting drought resilience in locally adapted sweet potato genotypes. To achieve this aim, the study was conducted through the use of untargeted ultra-high performance liquid chromatography-mass spectrometry (UHPLC-MS) based metabolomic analysis, which includes multivariate data analysis, viz. principal component analysis (PCA), and orthogonal projection to latent structures discriminant analysis (O) PLS-DA. This research provides important guidance for breeding programs regarding key metabolites that sweet potato cultivars use in responding to drought, offering useful basic knowledge to improve the crop for targeted traits, including drought tolerance.

## 2. Materials and Methods

### 2.1. Sweet Potato Planting

Two sweet potato cultivars (Atacama and Blesbok) were selected for the drought experiments. Atacama has been indicated as drought-tolerant in early vegetative screening [22]. Blesbok is the most dominant commercial sweet potato cultivar in South Africa, also planted in neighboring countries and Honduras [24]. Characteristics of each cultivar are described in Supplementary Table S1. The trial was planted under a rainout shelter at the Agricultural Research Council-Vegetable, Industrial and Medicinal Plants Research Institute (ARC-VIMP), Roodeplaat Campus, Pretoria, South Africa (25.60° S, 28.345° E; 1189 m altitude) during the February 2024 growing season. Meteorological data recorded at the ARC–VIMP research site during the 2024 growing season is indicated in Supplementary Table S2. The rainout shelter was made of a corrugated polycarbonate sheet supported on steel profiles (pillars and rails) with a thickness of 1.25 mm and UV protected on both sides. Sweet potato cuttings were planted in bags with 40 kg of soil per bag, with the soil prepared according to normal cultivation practices (soil conditions are indicated in Supplementary Table S3) at a plot size of 242 m^2^, inter-spacing of 0.7 m, and intra-spacing of 0.4 m. The trial was laid out in a randomized block design, with each plot consisting of 12 plants. The area under the rainout shelter was covered with black plastic mulch before placing the bags for insulation and to also control weeds from growing. Plants were grown for five weeks before imposition of drought stress, with continuous irrigation to field capacity (FC). Three water regimes—30% (control, 70% FC), 50% (mild stress, 50% FC), and 70% (severe stress, 30% FC)—were applied, and each treatment and cultivar were replicated four times (Figure S1). A total of 576 plastic bags were used. Fertilizers (multifeed and LAN (28)), insecticides (Decis^®^from Agro Bayer, Isando, South Africa, Profenofos 500 EC, Biomectin (R)), and fungicides (Nanogreen SC and Azoxystrobin 250 SC) were applied to the soil according to the manufacturer’s instructions. Water management was carried out through monitoring of soil water content (every two days to establish the amount of water needed) by measuring the relative water content using the formula below [25].

$$RSWC=\frac{Current\;pot\;weight-soil\;dry\;weight}{weight\;of\;soil\;watered\;to\;field\;capacity\;wet}\;\times 100\%$$

### 2.2. Metabolite Extraction

Young leaves from plants under drought and non-drought stressed conditions (30%, 50% and 70%) were collected at two weeks post drought imposition, representing early drought stress. The collected plant leaf samples were kept in −80 °C prior to analysis. Leaves (200 mg) were individually ground with liquid nitrogen, and the homogenate was resuspended with 1.5 mL prechilled 80% methanol (−20 °C, HPLC grade, Minema Chemicals, Roodeport, South Africa) in 2 mL Eppendorf tubes, followed by good vortexing. The extraction process was carried out using the methodology outlined in [26] and Makhubu et al. [27]. The samples were centrifuged at 2850 revolutions per minute (rpm) for 5 min at 4 °C after being sonicated for 2 h in ice-cold water. The supernatant (extract) was transferred to a 2 mL Eppendorf tube and stored at 4 °C. Glass vials with 0.5 mL inserts (Alwsci Technologies, Hangzhou, China, 6 × 31 mm) were then filled with the supernatants after the contents had been filtered through 0.22 µm nylon filters. Each sample group had four replicates prepared, which were kept at 4 °C until further analysis.

### 2.3. UHPLC-ESI-MS Analysis

Using a liquid chromatography–quadrupole time-of-flight tandem mass spectrometer (LCMS-9030 qTOF, Shimadzu Corporation, Kyoto, Japan) at the University of Venda, Department of Biochemistry, leaf extracts were assessed for metabolites following the methodology outlined in [28]. Employing a Shim-pack Velox C18 column (100 mm × 2.1 mm, 2.7 µm particle size; Shimadzu Corporation, Kyoto, Japan), the chromatographic separation was carried out at 55 °C. A 13 min procedure with the following gradient conditions was used to analyze each sample (3 μL): solvent A was 0.1% formic acid in Milli-Q water (HPLC grade, Merck, Darmstadt, Germany); solvent B was methanol (UHPLC grade, Romil SpS, Cambridge, UK) mixed with 0.1% formic acid. Throughout the designated gradient, the flow rate was maintained at 0.45 mL/min under the following separation conditions: After 2 min of equilibration at 10% B, 10–60% B was induced during 3–5 min. From 5 to 8 min, the settings were adjusted from 60% to 90% B, and from 8 to 11 min, the gradient was maintained at 90%. After 1 min (11–12 min), the gradient was brought back to its starting 90–60%; shortly thereafter, there was a 1 min column equilibration pause. The qTOF high-definition mass spectrometer, which was configured for negative electrospray ionization for data acquisition, was used for chromatographic analysis. The set of settings included the following: heat block temperature (400 °C), detector voltage (1.8 kV), DL temperature (280 °C), interface voltage (−3 kV), interface temperature (300 °C), nebulization and dry gas flow (3 L/min), and flight tube temperature (42 °C). With argon serving as the impact gas and a collision energy of 30 eV, fragmentation experiments were carried out using a spread of 5 eV.

## 3. Data Analysis

### 3.1. Raw Data Pre-Processing

The raw data in negative electrospray ionization mode (ESI negative) obtained from the LCMS-9030 qTOF were extracted as mzML files and processed using XCMS online (version 3.7.1, http://XCMSOnline.scripps.edu/) (accessed on 13 April 2024). Data pre-processing was performed using XCMS with UPLC/UHD Q-TOF negative mode parameters following Ramabulana et al. [28] and Makhubu et al. [27], employing the CentWave feature detection method with a maximum threshold of 15 ppm, a signal-to-noise ratio of 6, prefilters set at an intensity of 700, peaks at 3, and noise set at 15. Retention time correction was performed using the obiwarp method with a profStep of 1. For alignment, the minimum fraction (minfrac) of samples was set to 0.5, and the width of overlapping *m*/*z* for peak density chromatograms and grouping across samples (mzwid) was set at 0.025 *m*/*z*. The Mann–Whitney non-parametric test was applied to assess differences between group means (Atacama and Blesbok, drought (50% and 30% FC) and non-drought treatments (70% FC) of the two cultivars), followed by post hoc analysis, with data normalization using median fold change.

### 3.2. Multivariate Data Analysis

The resulting feature table from XCMS included 4700 features from Atacama vs. Blesbok; 6895 features from Atacama 30%, 50% and 70%; 5537 features from 30% Atacama vs. 50% Atacama; 6079 features from 30% Atacama vs. 70% Atacama; 5756 features from 50% Atacama vs. 70%; 4239 features from Blesbok 30%, 50% and 70%; 3838 features from 30% Blesbok vs. 50% Blesbok; 3777 features from 30% Blesbok vs. 70% Blesbok; and 3957 features from 50% Blesbok vs. 70% Blesbok. These features were imported into SIMCA version 17.0 software (Sartorius, Umeå, Sweden), normalized, and Pareto scaled before applying the model. Both an unsupervised model, principal component analysis (PCA), and supervised orthogonal projections to latent structures discriminant analysis (OPLS-DA) were employed. S-plots from the OPLS-DA score plots were generated, and significant biomarkers with [p (corr)] ≥ 0.5 and covariance of (p1) ≥ 0.05 were annotated by matching their spectral features and retention times with databases, leading to their putative identification [29]. Venn diagram (version 2.1) was used to present the overlap of metabolites in the two cultivars at different drought regimes. Additionally, Variable Importance in Projection (VIP) was used for screening the three drought stress conditions in each cultivar. From the PLS-DA model, the VIP scores were generated. It is generally accepted that variables with VIP scores greater than 1.0 are typically considered significant, and this threshold is commonly used as the criterion for selecting important variables [30].

### 3.3. Metabolite Annotation and Pathway Analysis

Annotated metabolites were identified from untargeted UHPLC–MS data through a combination of accurate mass matching. Identification and annotation were performed by comparing observed *m*/*z* values and retention times to entries in publicly available metabolite databases, including the Global Natural Product Social Molecular Networking (GNPS), Human Metabolome Database (HMDB), Massbank, KNApSAcK, COCONUT, Foodb, Sirius, and PubChem library. Level 2 annotation confidence (putatively annotated compounds) was assigned based on spectral similarity without comparison to authentic standards. These annotated metabolites were then used for pathway analysis, mapping the metabolic processes influenced by the experimental conditions. The analysis was conducted using Metabolic Pathway Analysis integrated into the MetaboAnalyst toolset (version 6.0; http://www.metaboanalyst.ca/, accessesd on 5 May 2024), which maps pathways using established KEGG metabolic pathways. Compound names were used as input for pathway analysis, relative centrality was chosen to examine the topology of node importance (pathway impact), and a scatter plot was utilized for display (–log(*p*-value) (enrichment score). *Arabidopsis thaliana* (KEGG) was chosen as the path library; this was due to the absence of a curated KEGG pathway library for *Ipomoea batatas*. This integrative approach enabled the identification of key metabolic pathways significantly affected by drought stress, thus providing insight into cultivar-specific metabolic adaptation.

## 4. Results

### 4.1. Comparative Analysis of Metabolites Under Non-Drought Stress Conditions

Untargeted metabolomics was used to determine and analyze the metabolites in Atacama and Blesbok leaf samples under normal conditions. Figure S1 represents the VIP scores (Figure S1A) for metabolites discriminating between Atacama and Blesbok cultivars under normal (non-drought) conditions (red for high and blue for low intensities). The vertical axis lists metabolites, while the horizontal axis indicates their scores from PLS-DA, highlighting the metabolites most influential in differentiating the two cultivars. Higher VIP scores indicate greater importance in distinguishing between the cultivars. The VIP analysis has revealed that Atacama and Blesbok cultivars exhibit distinct metabolic profiles. Phenolic compounds, lipids, terpenoids, flavonoids, and amino acids were critical in differentiating the two cultivars, with Atacama displaying a broader metabolite spectrum. Although most metabolites were abundant in Atacama, as indicated by their high intensities in the VIP heatmap and the stacked column chart on relative intensities (Figure S1B), their log2fold change values were less than 1, as shown in Table 1. This suggests that relative to Blesbok, the levels of these metabolites were lower when normalized, resulting in a negative fold change. Isolariciresinol 9′-O-alpha-L-arabinofuranoside had the highest VIP scores, suggesting that this metabolite is critical in differentiating the cultivars. Other notable contributors included alpha-Tocotrienol, Octadecyl ferulic acid, and Lupeol. Interestingly, among all metabolites, tricin 7-neohesperidoside (VIP = 2.01) and gibberellin A23 (VIP = 2.22) exhibited the highest log2fold changes (8.71 and 11.62, respectively) and showed high intensities in Blesbok.

### 4.2. Metabolic Differences Between Sweet Potato Cultivars Under Drought Stress

**Figure 1:**
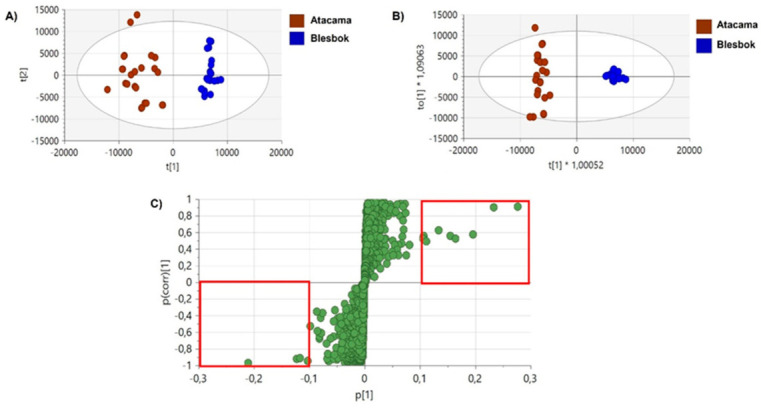
Unsupervised and supervised exploratory statistical analysis of Atacama and Blesbok under drought and non-drought stressed conditions. (**A**) Principal component (PC) scores for Atacama vs. Blesbok scatter plot of the Pareto-scaled data set. The quality parameters of the model were explained: variation/goodness of fit: R^2^X(cum) = 0.612 and Q^2^(cum) = 0.432. (**B**) An orthogonal projection to latent structures discriminant analysis (OPLS-DA) model. OPLS-DA: 1 + 1 + 0 component model. The quality parameters of the model were explained: variation/goodness of fit resulted in R^2^X(cum) = 0.372 and Q^2^(cum) = 0.974. (**C**) A loadings S-plot, with statistically significant features described to have (p (corr)) of ≥0.5 and covariance of (p1) ≥ 0.05. Up-regulated metabolites are highlighted in red box towards right, and the down-regulated metabolites are highlighted in red box towards left.

To understand how Blesbok and Atacama sweet potato cultivars performed under three drought stress conditions (30%, 50%, and 70%), PCA and OPLS-DA plots were computed. The PCA plot comparing Blesbok and Atacama reveals two distinct clusters, indicating that the metabolic profiles of these cultivars differ significantly (Figure 1A). The clear separation of Atacama and Blesbok samples in the OPLS-DA model further confirms that their metabolite profiles are distinctly different (Figure 1B). To gain more insight into the metabolic differences between Atacama and Blesbok, differential metabolite screening was performed, and the metabolites contributing to the distinct clustering were annotated. Among the 4700 identified metabolites, 10 key metabolites were significantly regulated based on S-plot loadings: 7 were up-regulated, and 3 were down-regulated (Figure 1C).

The regulated metabolites identified on Atacama and Blesbok cultivars are highlighted in Table 2 together with their molecular characteristics, fold changes, VIP scores, statistical significance, and classification into distinct metabolite classes. The relative stress levels of each metabolite were compared to those at 30% drought (70% FC). The criteria used for log2fold change were: very high, >4; high, 3–4; moderate, 1–2; decreased, <1. Of the 10 metabolites, kaempferol-3-O-galactoside (3.48) (*p*-value =2.27 × 10^−10^), chlorogenic acid (3.34) (*p*-value = 4.09 × 10^−14^), glc-glc-octadecatrienoyl-sn-glycerol (3.14) (*p*-value = 3.55 × 10^−15^), and apigenin-7-O-β-d-neohesperidosides (2.71) (*p*-value = 1.70 × 10^−4^) showed the highest log2fold change values and the most significant *p*-values, indicating significant variations in their levels. The 9,12,15-Octadecatrienoic acid, 3-(hexopyranosyloxy)-2-hydroxypropyl ester, (9Z,12Z,15Z)- (1.07) (*p*-value = 1.42 × 10^−4^) was moderately regulated. Although trehalose 6-phosphate and luteolin-6-C-glucoside were up-regulated based on S-plot loadings, their log2fold change was less than 1, implying decreasing change.

**Table 1:** Regulated metabolites identified between Atacama and Blesbok sweet potato cultivars under non-drought-stressed conditions.

Compound Name	Experimental Mass (*m*/*z*)	Rt (min)	Molecular Formula	Log2Fold Change	VIP Value	*p*-Value	Class
Isolariciresinol 9′-O-alpha-L-arabinofuranoside	492.031	4.66	C_25_H_32_O_10_	−10.64	2.23	4.10 × 10^−4^	Lignan glycosides
alpha-Tocotrienol	423.040	4.65	C_29_H_44_O_2_	−17.95	2.23	4.10 × 10^−4^	Vitamin E derivatives
Octadecyl ferulic acid	445.022	4.67	C_28_H_46_O_4_	−11.84	2.22	4.10 × 10^−4^	Coumaric acids and derivatives
Lupeol	425.548	4.65	C_30_H_50_O	−7.66	2.21	4.10 × 10^−4^	Triterpenoid
10-Octacosene-1,12-diol	424.731	4.65	C_28_H_56_O_2_	−7.96	2.21	4.10 × 10^−4^	Fatty alcohol
Tricin 7-neohesperidoside	638.366	7.80	C_29_H_34_O_16_	8.71	2.01	5.54 × 10^−4^	Flavonoid-7-o-glycosides
Tryptophan	203.092	3.73	C_11_H_12_N_2_O_2_	−0.82	2.20	1.55 × 10^−4^	Indolyl carboxylic acids and derivatives
epsilon-Tocopherol	410.330	4.42	C_28_H_42_O_2_	−7.77	2.20	4.09 × 10^−4^	Vitamin E derivatives
Gibberellin A23	378.146	7.79	C_20_H_26_O_7_	11.63	2.22	4.09 × 10^−4^	c20-gibberellin 6-carboxylic acids
Peonidin 3-sambubioside 5-glucoside	758.35	6.91	C_33_H_41_O_20_	−11.28	2.20	4.09 × 10^−4^	Anthocyanidin-5-o-glycosides
Glc-Glc-octadecatrienoyl-sn-glycerol (isomer 2)	722.273	6.89	C_33_H_56_O_14_	−9.07	2.19	8.26 × 10^−4^	Glycolipids
PC(20:1(13Z)/22:0)	871.071	4.65	C_50_H_98_NO_8_P	−11.50	2.19	4.09 × 10^−4^	Glycerophospholipid

Rt: Retention time in minutes; VIP: Variable Importance in Projection. The criteria used for log2fold change were: very high, >4; high, 3–4; moderate, 1–2; decreased, <1. Variable.

**Table 2:** Regulated metabolites between Atacama and Blesbok sweet potato cultivars under drought-stressed conditions.

Metabolites	Experimental Mass (*m*/*z*)	Rt (min)	Molecular Formula	Log2Fold Change	*p*-Value
Glc-Glc-octadecatrienoyl-sn-glycerol	721.444	6.34	C_33_H_56_O_14_	**3.14**	3.55 × 10^−15^
Chlorogenic acid	353.142	3.17	C_16_H_18_O_9_	**3.34**	4.09 × 10^−14^
Luteolin-6-C-glucoside	447.253	8.61	C_21_H_20_O_11_	0.47	7.93 × 10^−5^
9,12,15-Octadecatrienoic acid, 3-(hexopyranosyloxy)-2-hydroxypropyl ester, (9Z,12Z,15Z)-	559.314	6.59	C_27_H_46_O_9_	1.07	1.42 × 10^−4^
Apigenin-7-O-β-d-neohesperidoside	577.339	6.98	C_27_H_30_O_14_	**2.71**	1.70 × 10^−4^
Trehalose 6-phosphate	421.237	8.81	C_12_H_23_O_14_P	0.48	1.77 × 10^−4^
Kaempferol-3-O-galactoside	447.314	8.58	C_21_H_20_O_11_	**3.48**	2.27 × 10^−10^
Isomangiferin	423.252	9.22	C_19_H_18_O_11_	−15.80	0
N,-p-Coumaroyl-N’-feruloylputrescine	409.024	4.38	C_23_H_26_N_2_O_5_	−5.40	4.44 × 10^−15^
Adenosine 5′-monophosphate	347.081	4.08	C_10_H_14_N_5_O_7_P	−9.18	1.19 × 10^−13^

Rt: Retention time in minutes. Only significant log2fold changes are highlighted in bold. The relative stress conditions of each metabolite were compared to 30% (70% FC) of drought. The criteria used for log2fold change were: very high, >4; high, 3–4; moderate, 1–2; decreased, <1.

**Figure 2:**
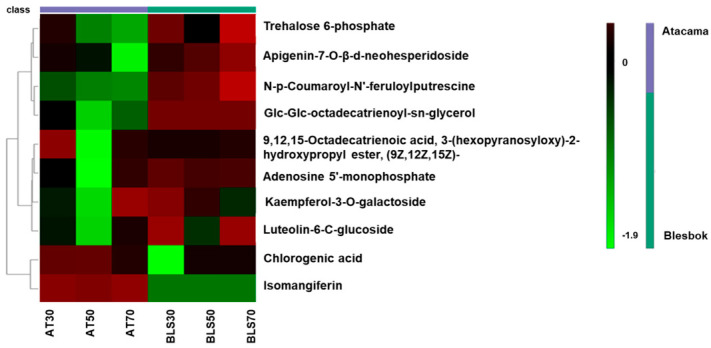
Heatmap depicting variations in metabolite concentrations between Atacama and Blesbok. Each annotated metabolite’s mean peak intensity is shown after data normalization and Pareto scaling. The color scheme in the legend indicates fold change increases (dark red to red), decreases (green), and significant differences between the cultivars. AT for Atacama and BLS for Blesbok.

A heatmap (Figure 2) was generated based on the results obtained from Table 2, which illustrates the concentration levels of metabolites in Atacama and Blesbok under different stress conditions (30%, 50%, and 70%). The results indicated that most metabolites in Blesbok showed increasing concentrations across the three conditions, except for isomangiferin, which decreased at all conditions, and chlorogenic acid, which only decreased at the 30% level. The significant metabolic changes in Blesbok could be due to its sensitivity, reflecting its enhanced ability to respond to drought stress, suggesting a robust survival mechanism under drought conditions. Conversely, most metabolites in Atacama displayed decreasing concentration levels, with some showing no significant response. The decreasing concentrations of metabolites in Atacama may be attributed to its inherent drought tolerance, reducing the need for extensive up-regulation of these compounds as a defensive mechanism. Notably, isomangiferin concentrations increased at all conditions in Atacama, while chlorogenic acid increased at the 50% and 70% conditions.

**Figure 3:**
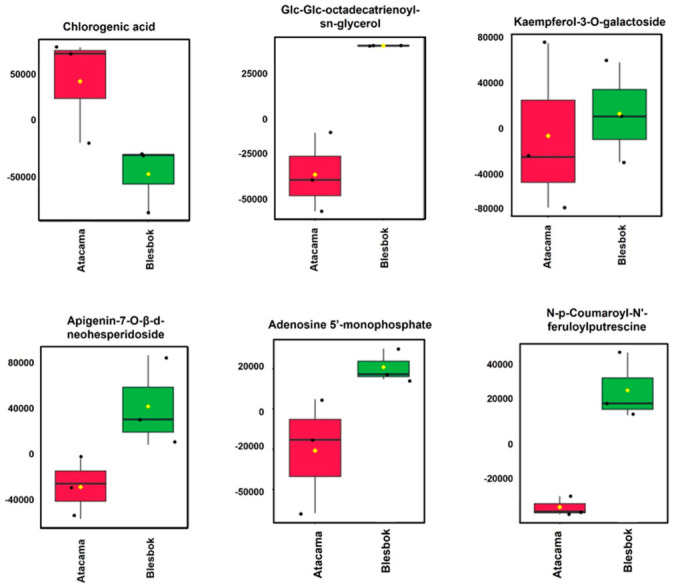
Box-and-whisker plots demonstrating the quantitative changes of metabolites, including the four highest log2fold change and two down-regulated metabolites. The mean value is represented by yellow dots, and each replicate is shown by black dots. Metabolite peak area quantification for Atacama is represented in red, while Blesbok is shown in green. Peak area quantification of metabolites extracted from the Atacama is shown in red, while peak area quantification of metabolites extracted from Blesbok is shown in green.

The regulation of this concentration at different drought stress conditions was further illustrated using a box-and-whisker plot (Figure 3), which highlighted the quantitative changes in key metabolites between the two cultivars. The plot features metabolites with high log2fold changes, including chlorogenic acid, glc-glc-octadecatrienoyl-sn-glycerol, apigenin-7-O-β-d-neohesperidoside, and kaempferol-3-O-galactoside. Additionally, the two significantly down-regulated metabolites, N-p-coumaroyl-N’-feruloylputrescine and adenosine 5′-monophosphate, were also quantified. All these metabolites were up-regulated in Blesbok, with the exception of chlorogenic acid, which was the only metabolite that was up-regulated in Atacama. Adenosine 5′-monophosphate appeared down-regulated in the S-plot, but both the box-and-whisker plot and heatmap showed its up-regulation and increasing concentrations in Blesbok.

To investigate how pathway topology was influenced by these metabolites, a KEGG pathway analysis was performed. Figure S3 summarizes the pathway analysis based on the annotated metabolites listed in Table 2. The analysis highlights zeatin biosynthesis (1) and starch and sucrose metabolism (2) as highly significant, indicated by their positions at the top of the *y*-axis. Key metabolites for these pathways include Adenosine 5′-monophosphate and trehalose phosphate, respectively. Additionally, phenylpropanoid, flavonoid biosynthesis, and purine biosynthesis (the latter with the largest node) pathways were found to have a significant influence.

### 4.3. Metabolic Variations Within Atacama and Blesbok in Response to Drought Stress

**Figure 4:**
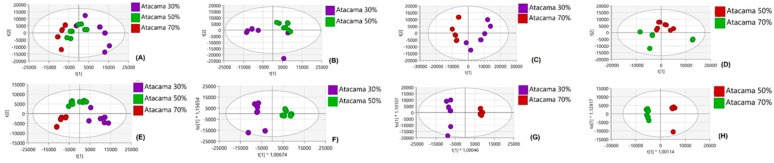
Unsupervised and supervised exploratory statistical analysis of Atacama under drought and non-drought stressed conditions. (**A**–**D**) PC scores for Atacama treatments scatter plot of the Pareto-scaled data set, I PLS-DA model of Atacama multigroup for 30%, 50% and 70%, (**F**–**H**) OPLS-DA score plots for comparing between two Atacama treatments. The quality parameters of the models were explained using variation/goodness of fit: (**A**) 5 component model, R^2^X(cum) = 0.723 and Q^2^(cum) = 0.353, (**B**) 5 component model, 0.816 and Q^2^(cum) = 0.406, (**C**) 3 component model, R^2^X(cum) = 0. 681 and Q^2^(cum) = 0.269, (**D**) 4 component model, R^2^X(cum) = 0.76 and Q^2^(cum) = 0.384, (**E**) 6 component model, R^2^X(cum) = 0. 728 and Q^2^(cum) = 0.908, (**F**) 1 + 6 + 0 component model, R^2^X(cum) = 0. 869 and Q^2^(cum) = 0.972, (**G**) 1 + 2 + 0 component, R^2^X(cum) = 0.594 and Q^2^(cum) = 0.934, (**H**) 1 + 2 + 0 component, R^2^X(cum) = 0. 487 and Q^2^(cum) = 0.951.

**Figure 5:**
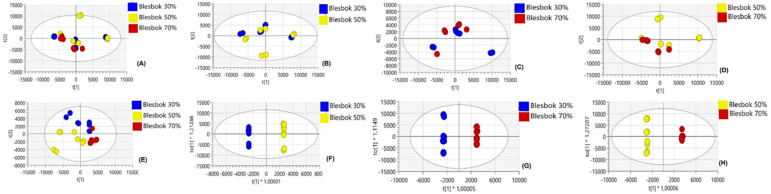
Unsupervised and supervised exploratory statistical analysis of Blesbok under drought and non-drought stressed conditions. (**A**–**D**) PC scores for Blesbok treatments scatter plot of the Pareto-scaled data set, (**E**) PLS-DA model of Blesbok multigroup for 30%, 50% and 70%, (**F**–**H**) OPLS-DA score plots for comparing between two treatments in Blesbok. The quality parameters of the models were explained using variation/goodness of fit: (**A**) 5 component model, R^2^X(cum) = 0.697 and Q^2^(cum) = 0.386, (**B**) 3 component model, R^2^X(cum) = 0.619 and Q^2^(cum) = 0.368, (**C**) 3 component model, R^2^X(cum) = 0.628 and Q^2^(cum) = 0.13, (**D**) 4 component model, R^2^X(cum) = 0.741 and Q^2^(cum) = 0.441, (**E**) 7 component model, R^2^X(cum) = 0.709 and Q^2^(cum) = 0.989, (**F**) 1 + 7 + 0 component model, R^2^X(cum) = 0.875 and Q^2^(cum) = 0.818, (**G**) 1 + 5 + 0 component model, R^2^X(cum) = 0.767 and Q^2^(cum) = 0.844, (**H**) 1 + 5 + 0 component model, R^2^X(cum) = 0.801 and Q^2^(cum) = 0.912.

Since these two cultivars exhibit distinct metabolic responses, as observed from the comparative analysis under normal conditions and between the two cultivars, we further wanted to compare drought stress conditions for each cultivar. Multigroup comparisons across regimes at 30%, 50%, and 70% drought stress levels, as well as pairwise comparisons: control (30%) vs. 50%, 30% vs. 70%, and 50% vs. 70%, were performed. Figure 4 illustrates how these metabolites vary at all stress conditions in Atacama, shown by Atacama 30% (purple), Atacama 50% (green), and Atacama 70% (red). The PCA plot comparing Atacama treatments (Figure 4A–D) revealed distinct clustering between treatments, and this is further shown in PLS-DA and OPLS-DA in Figure 4E–H. The different clustering in Atacama treatments showed that the different drought stress levels occupy distinct regions of the PCA score plot. Looking at Blesbok treatments, Figure 5 illustrates how these metabolites vary in all stress conditions in Blesbok, shown by Blesbok 30% (blue), Blesbok 50% (yellow), and Blesbok 70% (red). PCA plots also revealed clear separations of treatments in all stress conditions (Figure 5A–D). Furthermore, in PLS-DA and OPLS-DA plots, a clear separation was observed, suggesting significant metabolic differences in Blesbok (Figure 5E–H). Although the metabolite response in PLS-DA is not very clear, it does show the separation in the three treatments.

Table 3 details the metabolites and their log2fold changes based on the S-plot loadings from Blesbok and Atacama when compared to the untreated control. Our findings revealed that most metabolites were positively correlated with Atacama as compared to Blesbok. The predominant classes of regulated metabolites were flavonoid glycosides, fatty acids, and glycolipids. Notable up-regulation was observed in these classes, particularly in Atacama, although some metabolites were also significantly up-regulated in Blesbok. For example, chlorogenic acid was up-regulated in Atacama at 70% stress but down-regulated at 50%, whereas in Blesbok, it was up-regulated at 50% and down-regulated at 70%. Additionally, metabolites like dicaffeoylquinic acid and glc-glc-octadecatrienoyl-sn-glycerol were down-regulated in Atacama but not detected in Blesbok. Conversely, kaempferol-3-O-glucoside was up-regulated in Blesbok but not detected in Atacama. Apigenin-7-O-β-d-neohesperidoside showed significant up-regulation in Atacama at both 50% (5.27 log2fold) and 70% (3.31 log2 fold) stress conditions, while in Blesbok, it had minimal changes (0.29 log2 fold at 50% and 0.75 log2fold at 70%).

### 4.4. Pathway Analysis of Metabolites Under Drought Stress

**Figure 6:**
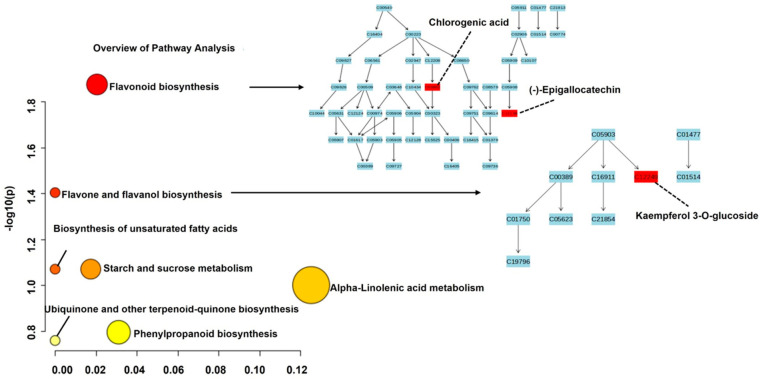
MetaboAnalyst (MetPA)-computed pathway analysis. Pathway impact values are plotted along the *x*-axis to reflect pathway topology analysis, while pathways are sorted along the *y*-axis to indicate pathway enrichment analysis based on their significance (*p*-value). Each pathway node’s color represents its *p*-value, with red denoting the lowest *p*-value and highest level of statistical significance. The pathway effect factor is represented by the node’s radius, where larger nodes have a greater influence.

The analysis highlights flavonoid biosynthesis (1) and flavone and flavanol biosynthesis (2) as highly significant metabolisms, indicated by their positions at the top of the *y*-axis (Figure 6). Key metabolites for these pathways include chlorogenic acid, (-)-Epigallocatechin, and kaempferol-3-O-glucoside. Additionally, starch and sucrose metabolism, alpha-linolenic acid metabolism, and phenylpropanoid biosynthesis were found to have the largest nodes, implying significant influence.

Venn diagram (Figure S4A) illustrates the distribution of metabolites across three different drought conditions in Atacama, i.e., 30%, 50%, and 70%. A total of 6603 metabolites (96.2%) were common across all three conditions. Atacama 30% (blue) shows 35 unique metabolites, Atacama 50% (yellow) has 3 unique metabolites, and Atacama 70% (green) has 5 unique metabolites. About 146 metabolites (2.1%) are shared between Atacama 30% and Atacama 50%, 61 metabolites (0.9%) are shared between Atacama 50% and Atacama 70%, and 12 metabolites (0.2%) are shared between Atacama 30% and Atacama 70%. This suggests that the majority of the metabolites are stable across the different drought conditions, with only a small percentage of metabolites being unique to specific conditions. These unique metabolites were then presented in Figure S4B, VIP (Variable Importance in Projection) score plot from a Partial Least Squares Discriminant Analysis (PLS-DA), which highlights the important metabolites that differentiate Atacama under three different drought conditions. All the top 10 metabolites were highly increased in Atacama 30%, with kaempferol 7-O-neohesperidoside having the highest VIP score, followed by ajugose, thus indicating its significant abundance. The high intensities in 30% Atacama were also observed in the bar graph Figure S4C.

The Venn diagram for Blesbok, shown in Figure S5A, revealed that no distinct metabolites were identified across the three stress conditions. This observation is further supported by the VIP plot in Figure S5B; however, the accumulation of each metabolite showed differences across the three stress conditions. Most metabolites were highly increased under the 30% stress condition, with a slight increase under 50% stress. High intensities supporting the increase of 30% were observed in the bar graph Figure S5C. Notably, isovitexin-7-O-glucoside was the only metabolite to show a significant increase at 70% stress. These differences suggest that each cultivar responds uniquely to drought stress, likely due to variations in their genetic or metabolic pathways.

## 5. Discussion

Improving a plant’s ability to withstand drought can help address water deficit challenges, enhancing crop productivity and contributing to global food security. As a result, efforts to develop drought-tolerant crops are increasing to reduce agriculture’s vulnerability. The current study investigated the metabolomic responses of Atacama (white-fleshed with purple skin) and Blesbok (cream-fleshed with purple skin) under three drought conditions, aiming to better understand their stress adaptive mechanisms. The levels of secondary metabolites in plants are strongly influenced by growing conditions, particularly under stress, which significantly alters the metabolic pathways responsible for their production. In this study, under normal conditions, both cultivars exhibited unique and overlapping metabolites, with Atacama showing higher relative intensities for specific metabolites compared to Blesbok.

Flavonoids and phenolic compounds were the most detected metabolites in the two cultivars under normal conditions. Some metabolites not detected under non-stress conditions were regulated in response to drought stress, while others present at low levels were also regulated. In most studies, metabolites have been reported to be significantly higher in the drought-tolerant cultivars as compared to the drought-sensitive [3,20]. In one study, for example, Liu et al. [31] analyzed seven Chinese sweet potato cultivars with varying drought tolerance levels and reported distinct metabolic responses between tolerant and sensitive cultivars. Their findings indicated that drought-sensitive cultivars primarily responded to stress by up-regulating plant signal transduction pathways, whereas drought-tolerant cultivars focused on regulating flavonoid and carbohydrate biosynthesis/metabolism. This observation aligns with the current study, where most metabolites correlated strongly with Atacama, the drought-tolerant cultivar. Also, the high regulation observed in Blesbok in this study highlighted that its metabolic response to drought stress is associated with its susceptibility.

### 5.1. Polyphenolic Compounds as Key Regulators of Drought Stress

Polyphenolic compounds are a large group of secondary metabolites widely distributed in plants and can be categorized into two main subgroups: flavonoids and phenolic acids [32]. Among the many health benefits that sweet potatoes offer are flavonoids, which are important antioxidants and nutritional components [33]. Plants under drought experience oxidative stress, which results in the release of reactive oxidative stress (ROS). Several metabolites, including flavonoids and polyphenols, are natural compounds that enable plants to neutralize ROS [34]. Most identified metabolites in the current study belonged to flavonoid glycosides and polyphenols and may act as shielding compounds that protect the sweet potato plants from oxidative damage caused by ROS by slowing down oxidative degradation and scavenging free radicals [35,36]. Compounds such as epicatechin, kaempferol, and apigenin are well known for their antioxidant and protective roles in plants under abiotic stress, including drought. They help scavenge reactive oxygen species (ROS) generated during stress, protecting plant cells from oxidative damage [37]. In a study by Kourouma et al. [38] investigating the chemical composition of twenty-five sweet potato cultivars from production sites in China, strong positive correlations were revealed between antioxidant activities and total polyphenol and flavonoid contents, highlighting their key role as antioxidants. These findings align with those of the current study, which revealed that under normal conditions, most metabolites were predominantly flavonoids and phenolic compounds, underscoring the antioxidant potential of sweet potatoes. The higher levels of flavonoids, phenolics, and other metabolites observed in the drought-tolerant Atacama cultivar, despite both cultivars having similar purple skin and being analyzed under normal conditions, suggest that drought tolerance may be associated with an inherently elevated accumulation of phenolics and related metabolites, potentially as a preparatory or adaptive mechanism to mitigate stress.

Biosynthetic pathways such as phenylpropanoid, flavonoid, flavone, and flavanol biosynthesis were among the enriched pathways known to play a crucial role in regulating plant stress resistance. Phenylpropanoids play a critical role in enabling plants to withstand both biotic and abiotic challenges, contributing significantly to their overall stability [39]. Compounds that belong to the phenylpropanoid class often have a significant role in plant development and how plants interact with the environment [40,41]. Resistance to abiotic stress, in particular, is heavily reliant on the phenylpropanoid pathway, which is a key secondary metabolic process predominantly mediated by flavonoids and phenylpropanoids [42]. In this study, most metabolites were more abundant under mild stress compared to severe stress, with the highest log2fold changes observed in flavonoids. This aligns with findings by Althwab et al. [33], who reported that purple sweet potato is rich in polyphenols and flavonoids, further supporting the antioxidative potential of these compounds in the current study. Interestingly, while the high log2fold changes were predominantly observed under mild stress, Gharibi et al. [43] found that polyphenols such as luteolin-7-O-glycoside and 1,3-dicaffeoylquinic acid increased with prolonged drought stress in *Achillea pachycephala* Rech. f., this suggests that the accumulation of specific metabolites may vary depending on stress intensity and plant species. In addition, dicaffeoylquinic acid and chlorogenic acid were identified in the leaves of Atacama and Blesbok. Although their regulation differed between the moderate and severe stress conditions, these metabolites may have contributed to the flavonoid and phenylpropanoid metabolome pathways.

Chlorogenic acid is a derivative of caffeoylquinic acid, which is found in the highest concentrations in several sweet potatoes [44]. Chlorogenic acid has been previously quantified in sweet potato by Zheng and Clifford [45], demonstrating significant antioxidant activity and strong 1,1-diphenyl-2-picrylhydrazyl (DPPH) radical scavenging properties, along with other polyphenols found in purple sweet potato roots [46]. Chlorogenic acid plays a pivotal role in mitigating oxidative stress in plants through multiple antioxidative pathways, including hydrogen atom transfer (HAT), radical adduct formation (RAF), sequential proton loss electron transfer (SPLET), and single electron transfer–proton transfer (SET-PT). These mechanisms collectively enable chlorogenic acid to neutralize reactive oxygen species and maintain cellular redox balance under stress conditions [47]. Chlorogenic acid has been reported to accumulate in sweet potato under cold stress conditions [48]. Similarly, increased levels of chlorogenic acid have been observed in honeysuckle flower buds exposed to soil salinity [49]. Beyond abiotic stress responses, chlorogenic acid has also demonstrated strong bioactivity against pest attacks, including those affecting *Solanum melongena* L. [50], as well as other insect infestations [51,52]. Another flavonoid, apigenin-7-O-β-d-neohesperidoside (rhoifolin), was highly regulated in this study. The high regulation of this metabolite agrees with the findings of Santos et al. [53], which demonstrated that this metabolite, together with other flavonoids, was found to be considerably induced in citrus leaves under drought stress. The enhanced accumulation of flavonoids in sweet potato cultivars under drought stress in the current study is closely linked to their role in detoxifying harmful hydrogen peroxide (H_2_O_2_) molecules in the cytoplasm. This accumulation helps neutralize oxidative stress, with the subsequent oxidation of flavonoids being followed by their reconversion into primary metabolites through the action of ascorbic acid [54]. For this reason, as noted by Sharma et al. [42], the drought-induced accumulation of phenolic compounds primarily results from the modulation of the phenylpropanoid biosynthetic pathway, which is activated as part of the plant’s stress response mechanism. Additionally, rhoifolin has been profiled in *Jatropha integerrima* Jacq. extracts, showing high potential for antioxidant activities [55]. Several other studies have identified and quantified phenolic compounds from sweet potato that contribute to antioxidant activity [15,56,57]. Beyond their effects on sweet potato, our findings are partly consistent with those of Griesser et al. [58], who reported that polyphenols such as (−)-epicatechin, (−)-epicatechin gallate, kaempferol-3-O-glucoside, quercetin-3-O-glucoside, and quercetin-3-O-glucuronide significantly accumulated in grapevine leaves under prolonged drought stress. In contrast, these increases were not evident under short-term drought exposure, which was similarly observed in our study, where other cultivars exhibited early accumulation of these metabolites during initial drought stress, while others did not. This may indicate a delayed or cultivar-specific response that warrants further investigation under prolonged drought conditions. In this study, the profiled flavonoids in two cultivars under drought stress demonstrated free radical scavenging properties, reducing oxidative stress and protecting cells from drought-induced damage, suggesting that drought stress stimulates the accumulation of flavonoids in sweet potato.

### 5.2. The Role of Other Metabolites in Response to Drought Stress

Sugars also play an important role in many important biochemical and structural processes in plants, as well as acting as storage molecules. They further have links to other metabolic pathways as important metabolites and signaling intermediates [59]. The control of sugar metabolism, carbohydrate metabolism, and sugar transport may be impacted by the sugar accumulation caused by drought stress in plant organs [60]. Sucrose plays a crucial role in plant metabolism by serving as a substrate for biosynthetic processes, energy production, and the products of hydrolytic reactions. Additionally, it helps stabilize cellular membranes under stress conditions. Sucrose biosynthesis primarily occurs in the cytoplasm [61]. In the current study, only trehalose 6-phosphate, a disaccharide, was down-regulated in 50% Blesbok, but yet not identified in all other treatments. Although it was down-regulated, according to KEGG pathway analysis, sugar and sucrose metabolism were highly significant. Trehalose 6-phosphate is a key intermediate in trehalose biosynthesis, which acts as an important signaling metabolite that connects plant growth and development to its overall metabolic state [62]. It is also known to play a critical role in the regulation of sugar metabolism in plants and links their growth and development to their metabolic status [63,64]. The regulation of this metabolite in response to abiotic stress has been reported in salt-stressed maize [65] and drought-stressed rice [66]. This finding highlights the importance of trehalose biosynthesis in sweet potato’s stress response and suggests that further exploration of this pathway could offer insights into enhancing drought tolerance in sweet potato cultivars.

Adenosine 5′-monophosphate (AMP) is a purine nucleotide that serves as an important signaling molecule in plants, playing a role in various processes, including growth, development, and stress responses [67]. In this study, AMP overall performance, as shown by the pathway analysis, has contributed to zeatin biosynthesis, which was the highest significant biosynthesis, and has also contributed to the purine biosynthesis. These findings align with those of Shu et al. [68], who reported the overlapping of AMP between drought-susceptible and drought-tolerant cultivars in tomatoes. Moreover, AMP levels were significantly increased in *Haloxylon ammodendron* and *Haloxylon persicum* under drought conditions [69]. This suggests that AMP’s regulatory role in zeatin and purine biosynthesis might contribute to a plant’s ability to adapt to drought stress by modulating signaling pathways involved in stress tolerance and recovery, highlighting its potential as a target for improving drought tolerance in crops.

The identified metabolites, including flavonoids, phenolic acids, amino acids, glycolipids, and sugars, represent a critical metabolic pathway related to drought tolerance, which offers a valuable early indicator for selecting plants that can withstand drought stress. They also hold potential for integration into marker-assisted selection (MAS) programs to foster the development of drought-tolerant sweet potato varieties. This would streamline breeding processes and enhance efficiency. Moreover, combining metabolomic data with traditional breeding or applying cutting-edge biotechnological techniques such as CRISPR could allow for precise manipulation of stress-related pathways, resulting in more drought-resilient crops. This strategy would directly address food security challenges by boosting sweet potato production in drought-prone areas, ensuring a consistent food supply.

The results from the current study highlight significant differences in how Atacama and Blesbok respond to early drought stress at the metabolic level, likely driven by variations in their genetic and metabolic pathways. Blesbok exhibited more pronounced metabolic shifts, potentially reflecting a sensitivity-driven stress response, while Atacama’s response appeared more stable. Despite these metabolic differences, early drought stress did not lead to visually observable changes in above-ground biomass, suggesting that the observed effects are primarily molecular rather than phenotypic. This indicates that metabolic adjustments in response to stress might not always translate into visible changes in plant appearance. Although the study only focused on early drought stress, this still underscores the importance of molecular-level analyses in understanding drought tolerance. The high regulation of metabolites belonging to classes such as flavonoids, glycolipids, and sugars, specifically: chlorogenic acid, isomangiferin, apigenin-7-O-β-d-neohesperidoside, kaempferol-3-O-galactoside, kaempferol 7-O-neohesperidoside, ajugose, 8-p-Hydroxybenzlyquecetin, PE (18:0/22:0), and adenosine 5′-monophosphate may serve as early biomarkers of drought response. While this study provides insight into flavonoid accumulation and other important metabolic pathways in response to drought, it primarily focuses on the early stages of drought exposure.

## 6. Conclusions

Atacama and Blesbok clearly showed different metabolic adjustments under early drought stress, with key metabolite classes such as flavonoids, sugars, and glycolipids identified as potential biomarkers. These metabolites may also contribute to plant responses under other environmental stresses, underscoring their broader significance in stress adaptation and resilience. Although no visible phenotypic differences were observed in the two cultivars when exposed to drought, molecular differences highlight the value of metabolomics in uncovering hidden stress responses and provide a foundation for breeding programs aimed at improving drought tolerance in sweet potato. Nonetheless, the focus on early drought in this study limits an overall understanding of the broader regulatory mechanisms active during prolonged or terminal stress. To gain a comprehensive view, future studies should investigate metabolic changes across different drought stages and genotypes, correlating these with physiological and morphological traits. We do acknowledge that untargeted metabolomics, despite its comprehensive coverage, has inherent limitations in compound specificity, particularly when differentiating isomers or structurally similar metabolites without the use of authentic standards. To overcome some limitations of untargeted metabolomics, targeted approaches with authentic standards and optimized extraction methods could improve specificity and quantification. This approach would create a clearer picture of drought tolerance mechanisms and guide breeding programs aimed at improving sweet potato’s drought tolerance. Overall, leveraging these insights can enhance sweet potato resilience and support food security in regions affected by climate variability and water scarcity.

## Figures and Tables

**Table 3:** Up- and down-regulated metabolites in Atacama and Blesbok at 50 and 70% drought stress conditions and their log2fold changes.

Metabolite	EM (*m*/*z*)	Rt (min)	Molecular Formula	Adduct	Class	Cultivars Log2Fold Changes			
						Atacama 50%	Atacama 70%	Blesbok 50%	Blesbok 70%
Apigenin-7-O-β-d-neohesperidoside	577.339	6.80	C_27_H_30_O_14_	[M − H]^−^	Flavonoid glycoside	**5.27**	**3.31**	0.29	0.75
Quercetin 3-O-malonylglucoside	549.285	7.47	C_24_H_22_O_15_	[M − H]^−^	Flavonoid glycoside	**2.39**	−5.51	ND	ND
9,12,15-Octadecatrienoic acid, 3-(hexopyranosyloxy)-2-hydroxypropyl ester, (9Z,12Z,15Z)-	559.314	7.45	C_27_H_46_O_9_	M + FA − H	Fatty acid	1.17	1.97	−0.14	ND
Glc-Glc-octadecatrienoyl-sn-glycerol	721.366	6.91	C_33_H_56_O_14_	M + FA − H	Glycolipids	1.20	1.66	ND	ND
Dicaffeoylquinic acid	561.258	7.48	C_25_H_24_O_12_	M + FA − H	Phenolic acids	−2.35	ND	ND	ND
Isovitexin 7-O-glucoside	593.274	6.81	C_27_H_30_O_15_	[M − H]^−^	Flavonoid glycoside	−1.75	ND	ND	ND
Chlorogenic acid	353.088	3.16	C_16_H_18_O_9_	[M − H]^−^	Polyphenols	−0.36	1.95	0.23	−0.39
Isomangiferin	423.252	9.22	C_19_H_18_O_11_	[M + H]^−^	Xanthones	ND	**2.32**	−0.38	0.47
(-)-Epigallocatechin	305.143	3.51	C_15_H_14_O_7_	[M − H]^−^	Flavonoids	ND	ND	−0.38	ND
Glutamyltyrosine	309.207	5.44	C_14_H_18_N_2_O_6_	[M − H]^−^	Dipeptides	ND	ND	2.76	ND
Kaempferol-3-O-glucoside	447.253	7.88	C_21_H_20_O_11_	[M − H]^−^	Flavonoid glycoside	ND	ND	0.15	0.15
Trehalose 6-phosphate	421.237	8.00	C_12_H_23_O_14_P	[M − H]^−^	Disaccharide phosphate	ND	ND	−0.26	ND
Menatetrenone	445.237	7.43	C_31_H_40_O_2_	[M + H]^−^	Menaquinones	ND	ND	−0.16	ND
LysoPC (15:0)	481.258	7.62	C_23_H_48_NO_7_P	[M − H]^−^	Glycerophosphocholines	ND	ND	ND	−0.76
1,3-Dicaffeoylquinic acid	515.121	3.81	C_25_H_24_O_12_	[M − H]^−^	Quinic acids and derivatives	ND	ND	ND	0.24

EM: Experimental mass per charge; Rt: Retention time in minutes; ND: Not determined. Only significant log2fold changes are highlighted in bold. The relative stress conditions of each metabolite were compared to 30% (70% FC) of drought. The criteria used for log2fold change were: very high, >4; high, 3–4; moderate, 1–2; decreased, <1.

## Data Availability

The data is contained within the article. The raw datasets generated are available from the corresponding author upon request.

## Supplementary Materials

The following supporting information can be downloaded at: https://www.mdpi.com/article/10.3390/plants14223532/s1. Supplementary Figure S1: Variable importance in projection (VIP) score plot highlighting the most significant metabolites contributing to the differentiation between Atacama under 30% (non-stressed). The color scale represents the relative abundance of each metabolite across the stress conditions, with red indicating high levels and blue indicating low levels (A). Stacked column chart illustrates the relative intensities (in percentages) of metabolites profiled in the Atacama and Blesbok cultivars under non-drought-stressed conditions. The chart highlights the contribution of each metabolite to the overall metabolic profile (B). Supplementary Figure S2. The image presents a comparative visual assessment of above-ground parts of sweet potato cultivars, Atacama and Blesbok, subjected to drought stress at three stress conditions: 30%, 50%, and 70%. Each row represents one cultivar, with Atacama displayed in the top row and Blesbok in the bottom row. The columns depict the response at varying drought levels, moving from 30% (left) to 50% (middle) and 70% (right). Supplementary Figure S3. MetaboAnalyst (MetPA)-computed pathway analysis. Pathway impact values are plotted along the *x*-axis to reflect pathway topology analysis, while pathways are sorted along the *y*-axis to indicate pathway enrichment analysis based on their significance (*p*-value). Each pathway node’s color represents its *p*-value, with red denoting the lowest *p*-value and highest level of statistical significance. The pathway effect factor is represented by the node’s radius, where larger nodes have a greater influence. Supplementary Figure S4. Venn diagram illustrating the shared and unique metabolites in the Atacama cultivar under 30%, 50%, and 70% drought stress conditions. The numbers inside the circles represent metabolites unique to or shared between the stress conditions, with 30% Atacama (purple), 50% Atacama (yellow), and 70% Atacama (green) (A). VIP score plot highlighting the most significant metabolites contributing to the differentiation between Atacama under 30%, 50%, and 70% drought stress conditions. The color scale represents the relative abundance of each metabolite across the stress conditions, with red indicating high levels and blue indicating low levels (B). The bar graph provides a comparative analysis of the relative intensities of specific metabolites detected in the Atacama sweet potato cultivar under three drought stress conditions: 30%, 50%, and 70% stress conditions. Each bar represents the abundance of a particular metabolite at the corresponding stress condition, with red bars indicating 30% stress, purple bars indicating 50% stress, and orange bars indicating 70% stress (C). Supplementary Figure S5. Venn diagram illustrating the shared and unique metabolites in the Blesbok cultivar under 30%, 50%, and 70% drought stress conditions. The numbers inside the circles represent metabolites unique to or shared between the stress conditions, with 30% Blesbok (purple), 50% Blesbok (yellow), and 70% Blesbok (green) (A). VIP score plot highlighting the most significant metabolites contributing to the differentiation between Blesbok under 30%, 50%, and 70% drought stress conditions. The color scale represents the relative abundance of each metabolite across the stress conditions, with red indicating high levels, light yellow representing metabolites with an intermediate abundance, and blue indicating low levels (B). The bar graph highlights the relative intensities of specific metabolites detected in the Blesbok cultivar under three drought stress conditions: 30%, 50%, and 70%. Each bar represents the abundance of a particular metabolite at the corresponding stress condition, with orange bars indicating 30% stress, blue bars indicating 50% stress, and green bars indicating 70% stress (C). Supplementary Table S1: Key morphological traits, stress tolerance, and yield characteristics of Atacama and Blesbok sweet potato cultivars; Supplementary Table S2: Meteorological data recorded during the 2024 sweet potato growing season at the ARC–VIMP in Roodeplaat, South Africa; Supplementary Table S3. Soil conditions used for the study site.
