# Supplementary material for: Biochemical Responses of Atacama and Blesbok Sweet Potato (Ipomoea batatas L.) Cultivars to Early Drought Stress
Source: Plants (Basel). 2025 Nov 19;14(22):3532. doi: 10.3390/plants14223532 (PMC12655927; doi:10.3390/plants14223532)
Supplement: Supplementary file 1 [file plants-14-03532-s001.zip › plants-3914081-supplementary.pdf]

Supplementary Material

1 Supplementary Figures and Tables

1.1 Supplementary Figures

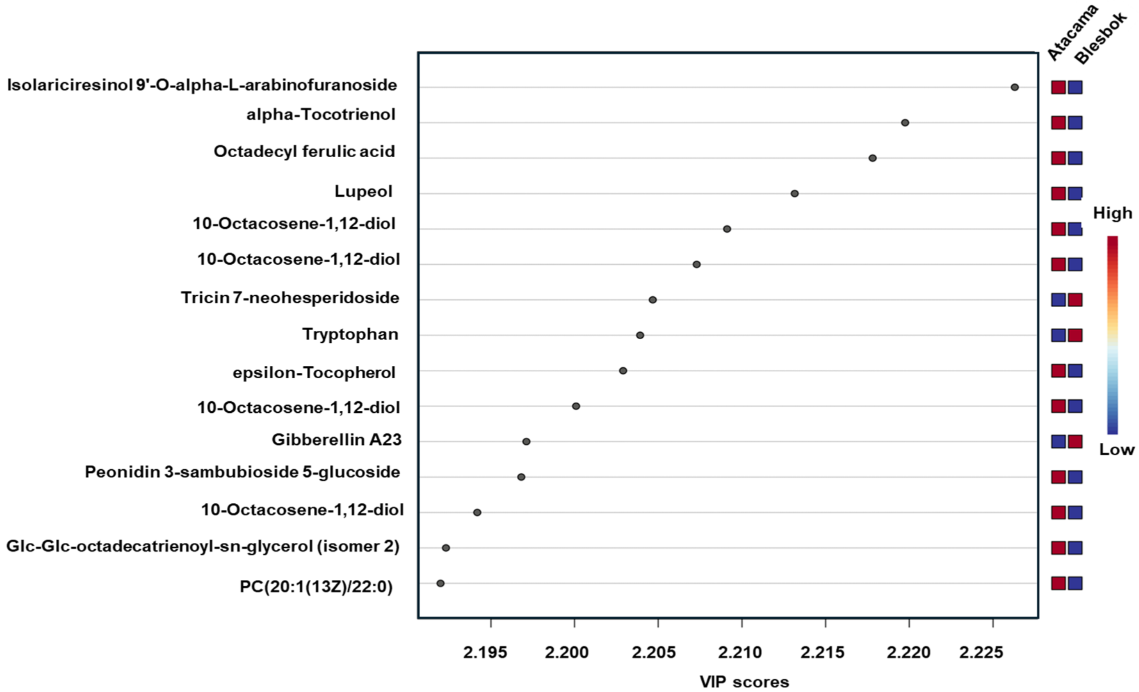

(A)

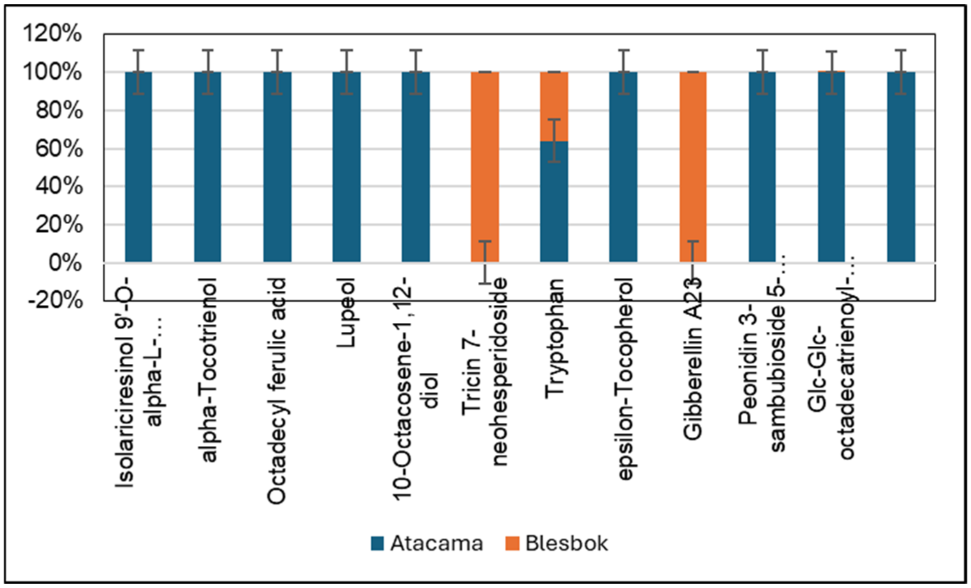

(B)

Supplementary Figure S1: Variable importance in projection (VIP) score plot highlighting the most significant metabolites contributing to the differentiation between Atacama under 30% (non-stressed). The color scale represents the relative abundance of each metabolite across the stress conditions, with

red indicating high levels and blue indicating low levels **(A)**. Stacked column chart illustrates the relative intensities (in percentages) of metabolites profiled in the Atacama and Blesbok cultivars under non-drought-stressed conditions. The chart highlights the contribution of each metabolite to the overall metabolic profile **(B)**.

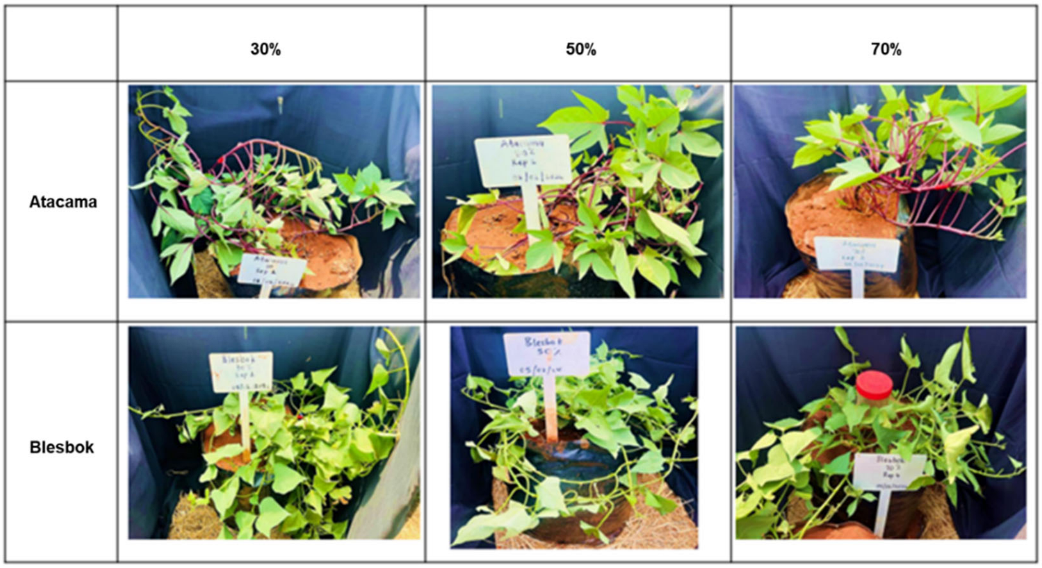

Supplementary Figure S2. The image presents a comparative visual assessment of above-ground parts of sweet potato cultivars, Atacama and Blesbok, subjected to drought stress at three stress conditions: 30%, 50%, and 70%. Each row represents one cultivar, with Atacama displayed in the top row and Blesbok in the bottom row. The columns depict the response at varying drought levels, moving from 30% (left) to 50% (middle) and 70% (right).

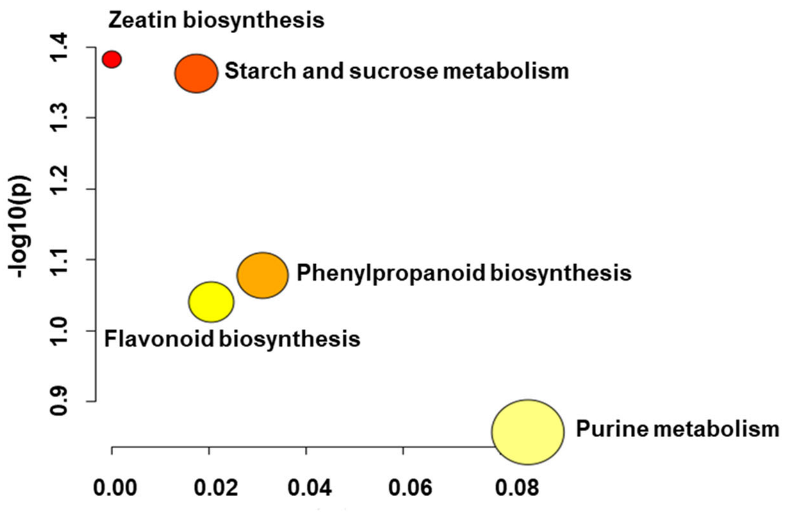

Supplementary Figure S3. MetaboAnalyst (MetPA)-computed pathway analysis. Pathway impact values are plotted along the x-axis to reflect pathway topology analysis, while pathways are sorted along the y-axis to indicate pathway enrichment analysis based on their significance (p-value). Each pathway node's color represents its p-value, with red denoting the lowest p-value and highest level of statistical significance. The pathway effect factor is represented by the node's radius, where larger nodes have a greater influence.

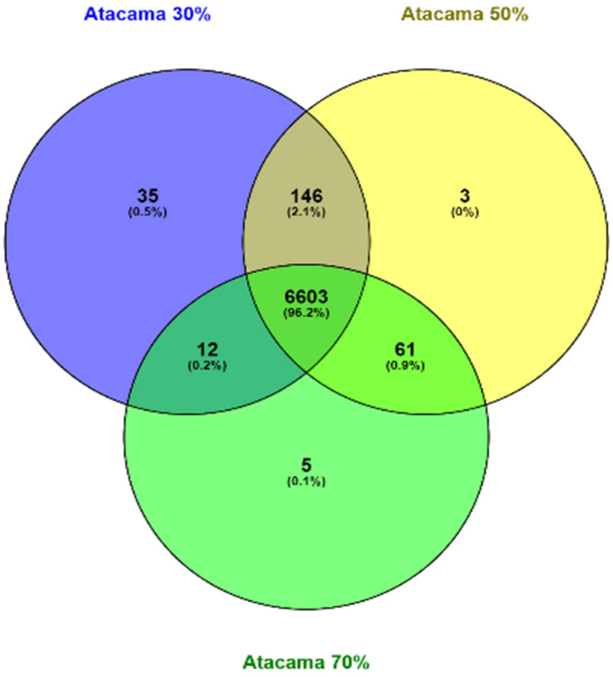
Atacama 30%
Atacama 50%
Atacama 70%
35
(0.5%)
146
(2.1%)
3
(0%)
6603
(96.2%)
12
(0.2%)
61
(0.9%)
5
(0.1%)

(A)

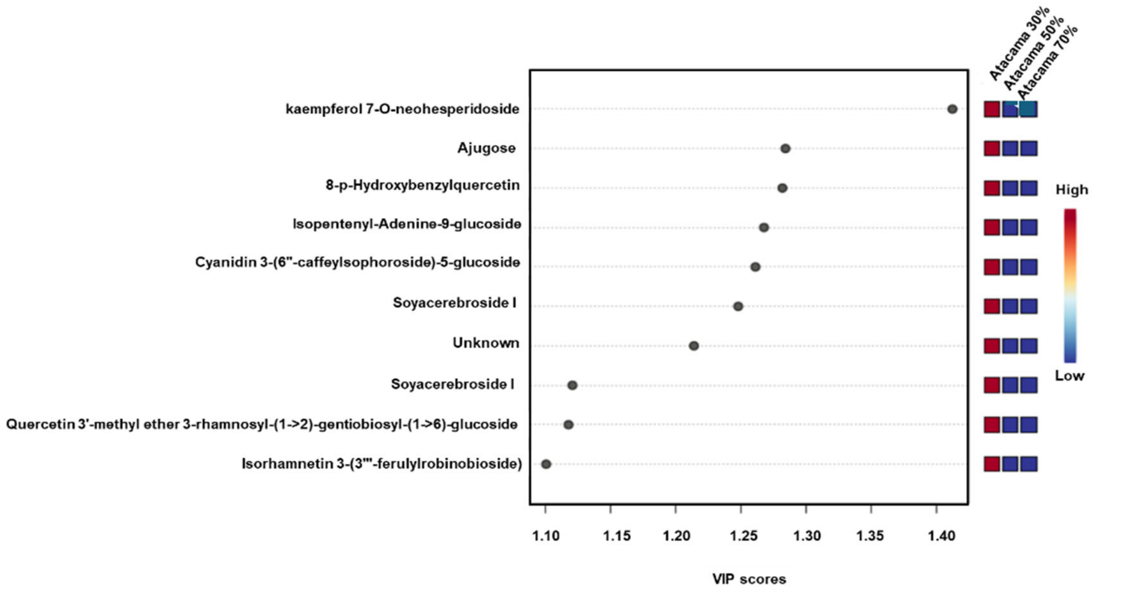
Atacama 30%
Atacama 50%
Atacama 70%
kaempferol 7-O-neohesperidoside
Ajugose
8-p-Hydroxybenzylquercetin
Isopentenyl-Adenine-9-glucoside
Cyanidin 3-(6''-caffeylsophoroside)-5-glucoside
Soyacerebroside I
Unknown
Soyacerebroside I
Quercetin 3'-methyl ether 3-rhamnosyl-(1->2)-gentiobiosyl-(1->6)-glucoside
Isorhamnetin 3-(3'''-ferulylrobinobioside)
High
Low
1.10
1.15
1.20
1.25
1.30
1.35
1.40
VIP scores

(B)

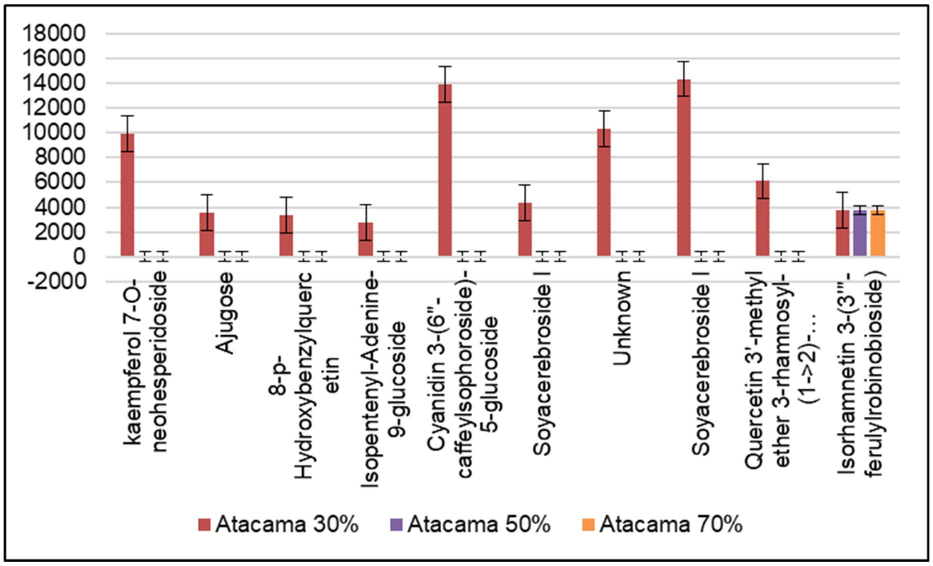

(C)

Supplementary Figure S4. Venn diagram illustrating the shared and unique metabolites in the Atacama cultivar under 30%, 50%, and 70% drought stress conditions. The numbers inside the circles represent metabolites unique to or shared between the stress conditions, with 30% Atacama (purple), 50% Atacama (yellow) and 70% Atacama (green) **(A)**. VIP score plot highlighting the most significant metabolites contributing to the differentiation between Atacama under 30%, 50%, and 70% drought stress conditions. The color scale represents the relative abundance of each metabolite across the stress conditions, with red indicating high levels and blue indicating low levels **(B)**. The bar graph provides a comparative analysis of the relative intensities of specific metabolites detected in the Atacama sweet potato cultivar under three drought stress conditions: 30%, 50%, and 70% stress conditions. Each bar represents the abundance of a particular metabolite at the corresponding stress condition, with red bars indicating 30% stress, purple bars indicating 50% stress, and orange bars indicating 70% stress **(C)**.

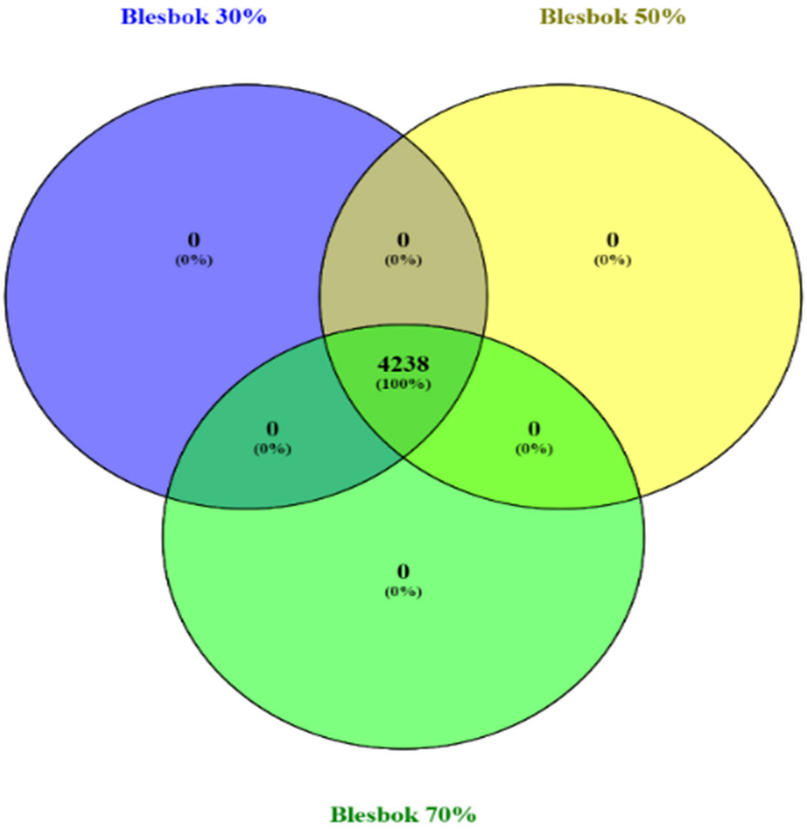

Blesbok 30%
Blesbok 50%
Blesbok 70%
0
(0%)
0
(0%)
0
(0%)
4238
(100%)
0
(0%)
0
(0%)
0
(0%)

(A)

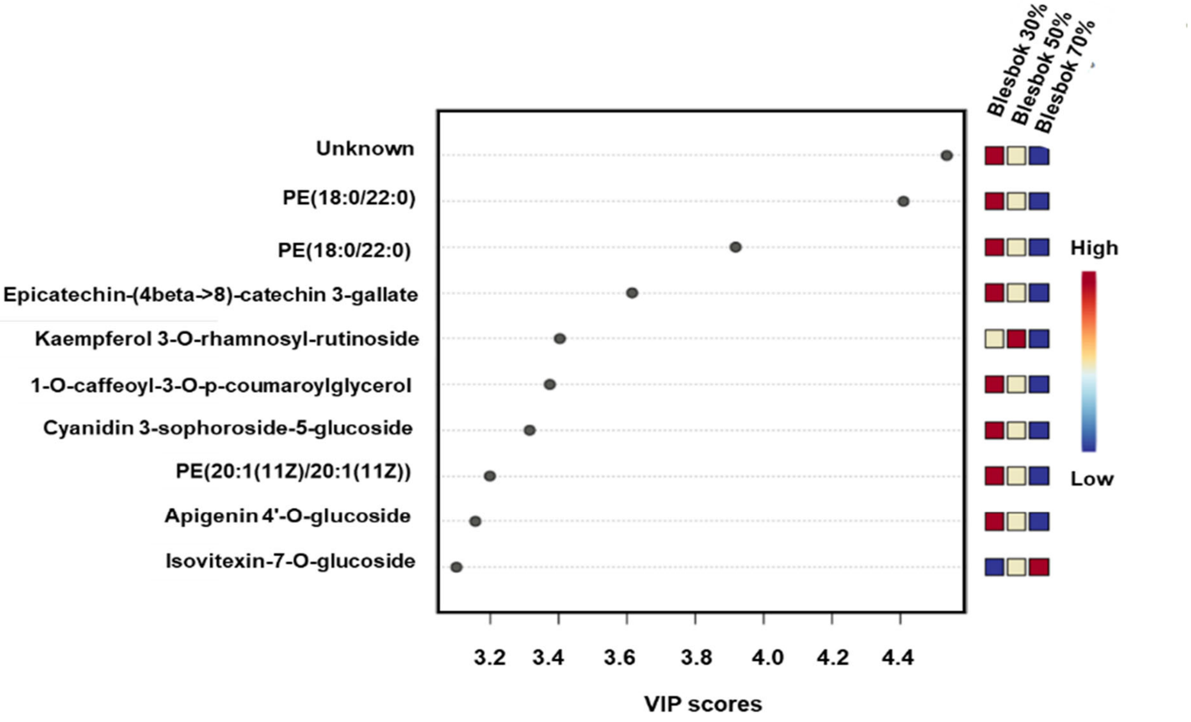

Blesbok 30%
Blesbok 50%
Blesbok 70%
Unknown
PE(18:0/22:0)
PE(18:0/22:0)
Epicatechin-(4beta->8)-catechin 3-gallate
Kaempferol 3-O-rhamnosyl-rutinoside
1-O-caffeoyl-3-O-p-coumaroylglycerol
Cyanidin 3-sophoroside-5-glucoside
PE(20:1(11Z)/20:1(11Z))
Apigenin 4'-O-glucoside
Isovitexin-7-O-glucoside
High
Low
3.2
3.4
3.6
3.8
4.0
4.2
4.4
VIP scores

(B)

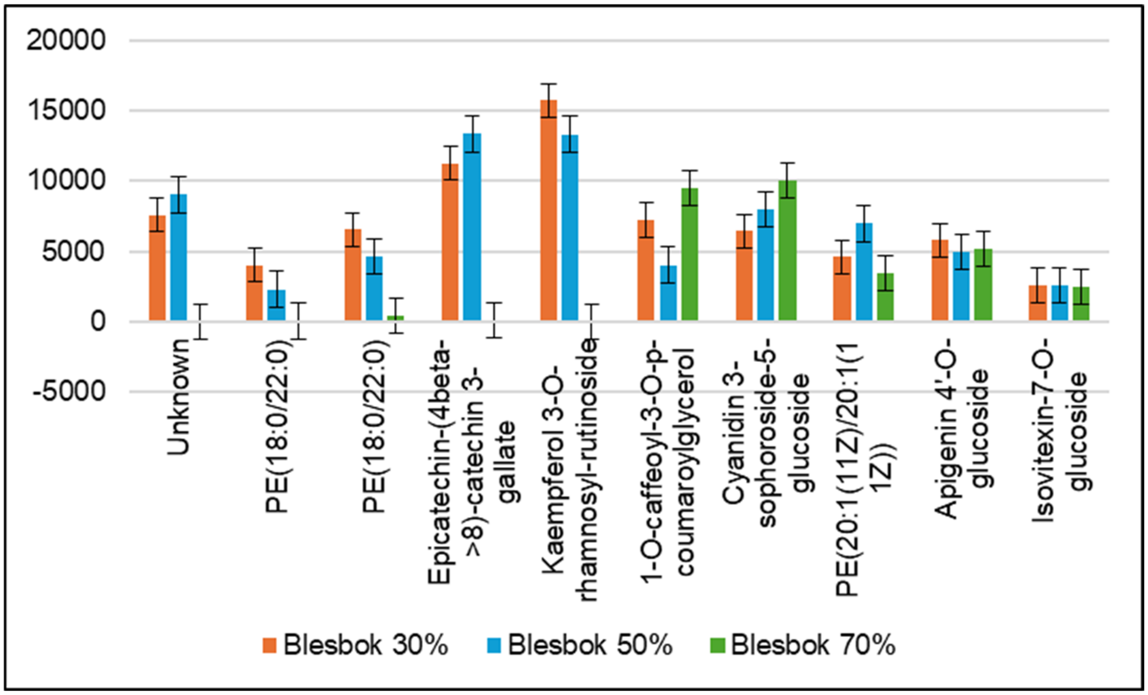

(C)

Supplementary Figure S5. Venn diagram illustrating the shared and unique metabolites in the Blesbok cultivar under 30%, 50%, and 70% drought stress conditions. The numbers inside the circles represent metabolites unique to or shared between the stress conditions, with 30% Blesbok (purple), 50% Blesbok (yellow) and 70% Blesbok (green) **(A)**. VIP score plot highlighting the most significant metabolites contributing to the differentiation between Blesbok under 30%, 50%, and 70% drought stress conditions. The color scale represents the relative abundance of each metabolite across the stress conditions, with red indicating high levels, light yellow represents metabolites with an intermediate abundance and blue indicating low levels **(B)**. The bar graph highlights the relative intensities of specific metabolites detected in the Blesbok cultivar under three drought stress conditions: 30%, 50%, and 70%. Each bar represents the abundance of a particular metabolite at the corresponding stress condition, with orange bars indicating 30% stress, blue bars indicating 50% stress, and green bars indicating 70% stress **(C)**.

1.2 Supplementary Tables

Supplementary Table S1: Key morphological traits, stress tolerance, and yield characteristics of Atacama and Blesbok sweet potato cultivars.

**Cultivar**	**Origin**	**Breeding line**	**Pedigree**	**Characteristics**
Atacama	Peru		-	Root traits: white flesh; dark purple skin; round elliptic<br>Leaf trait: green foliage, purple petiole, lobed<br>Diseases/stress tolerance: susceptible to sweetpotato virus disease; medium drought tolerance<br>Pest tolerance: susceptible to *Cylas formicarius*<br>Yield: high dry mass, high yield
Blesbok	South Africa	1985-7-1	81-21-1204	Root traits: cream flesh; purple skin; long oblong<br>Leaf traits: green foliage; triangular<br>Diseases/stress tolerance: susceptible to potyviruses, *Fusarium* spp; Intermediate susceptible to drought<br>Yield: high yield, low dry mass; great storability

Supplementary Table S2: Meteorological data recorded during the 2024 sweet potato growing season at the ARC–VIMP in Roodeplaat, South Africa.

**Meteorological data (average values)**									
Month (2024)	Average Daily Maximum Temperature (°C)	Average Daily Minimum Temperature(°C)	Average Daily Maximum Relative Humidity (%)	Average Daily Minimum Relative Humidity (%)	Total Radiation (MJ/m2)	Average Wind Speed (ms)	Total Daily Rainfall (mm)	Total Relative Evapotranspiration (mm)	Vapour Pressure Deficit
January	30.76	15.98	93.59	33.9	21.26	0.9	4.51	4.46	1.05
February	32.33	16.67	92.47	30.71	22.81	0.81	2.73	4.86	1.3

Supplementary Table S3. Soil conditions used for the study site

**Analyte**	**Method**	**Units**	**Results**
Clay	3 Fractions	%	34
Sand	3 Fractions	%	56
Silt	3 Fractions	%	10
P	Bray-1	mg/kg	0.16
Na	Amm. Acetate	mg/kg	17.3
K	Amm. Acetate	mg/kg	94.9
Ca	Amm. Acetate	mg/kg	1180
Mg	Amm. Acetate	mg/kg	700
Electrical Conductivity	Saturated Paste Extraction	mS/m	94.8
pH	pH - Water	-	7.21
T SUUR	Extraction	cmoI/kg	0
Na me	Calculation	cmol/kg	0.0753
K me	Calculation	cmol/kg	0.2427
Ca me	Calculation	cmoI/kg	5.8882
Mg me	Calculation	cmol/kg	5.7613
Sv	Calculation	%	0
